# Development of Hyperuricemia and Hyperglycemia After Prolonged Consumption of Clams ( *Galatea paradoxa* ) at the Recommended Daily Allowance

**DOI:** 10.1002/fsn3.71357

**Published:** 2026-01-21

**Authors:** Samuel Adjei, Matilda Asante, Charles Mills‐Robertson, Samkeliso Takaidza, Vivash Naidoo, Perpetua Dagadu, Stephen Yormasah, Ruth Owu, George A. Asare

**Affiliations:** ^1^ Noguchi Memorial Institute of Medical Research (NMIMR), University of Ghana Accra Ghana; ^2^ Department of Dietetics University of Ghana Accra Ghana; ^3^ Department of Medical Laboratory Sciences University of Ghana Accra Ghana; ^4^ Department of Natural Sciences Vaal University of Technology Vanderbijlpark South Africa; ^5^ Department of Family Medicine University of the Witwatersrand Johannesburg South Africa; ^6^ Department of Pharmacology and Toxicology University of Ghana Medical School Accra Ghana

**Keywords:** clams, hyperglycemia, hyperuricemia

## Abstract

Hyperuricemia and hyperglycemia are growing global health concerns and early warning signs for serious chronic diseases such as gout, diabetes, and cardiovascular conditions. To explore whether long‐term consumption of clams influences these conditions, we conducted a 60‐day feeding study in rats. Fresh clams were boiled, dried, powdered, and administered orally to four groups (*n* = 7) at dosages 0 (control), 50, 150, and 250 mg/kg body weight. Results show the high‐dose group exhibited a significant reduction in liver weight compared to controls (*p* = 0.007). Biochemically, all clam‐fed groups displayed significant globulin levels and albumin/globulin ratios (*p* = 0.036). Total bilirubin levels were significantly lower in the low and medium dose groups relative to controls (*p* = 0.031 and *p* = 0.047, respectively). AST levels significantly differed between medium and high dose groups (*p* = 0.048). Additionally, TBA increased in a dose‐dependent manner, with the high‐dose group showing a marked rise (*p* = 0.0001). Renal function parameters remained largely unchanged except for uric acid, which increased in a clear dose‐dependent pattern: control 128.2 ± 52.7 μmol/L; low 145.1 ± 71.6; medium 161.6 ± 132.8; high 339.5 ± 169.7. These elevations were significant between controls and high‐dose (*p* = 0.018) and between low‐ and high‐dose groups (*p* = 0.037). Blood glucose also rose dose‐dependently, reaching 17.6 ± 4.9 mmol/L in the high‐dose group, significantly higher than all lower‐dose groups (*p* ≤ 0.015). The study underscores that shellfish's high purine content, such as in clams, may provoke hyperuricemia and hyperglycemia, especially at higher consumption levels.

## Graphical abstract

Prolonged consumption of clam, *Galatea paradoxa,* powder led to dose‐dependent increases in uric acid and blood glucose in rats. Liver weight decreased at the highest dose, accompanied by mild histological alterations. These findings suggest that high clam intake, even within recommended dietary levels, may contribute to metabolic disturbances associated with hyperuricemia and hyperglycaemia.

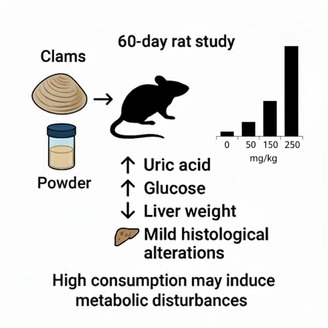

## 1 Introduction

Hyperuricemia, characterized by elevated uric acid levels, and hyperglycemia, are significant health concerns worldwide (Zhang 2025). These conditions serve as early indicators of chronic illnesses, such as gout, diabetes, and cardiovascular diseases, that significantly affect public health (Alsultan et al. 2025; Kuwabara et al. 2025). Therefore, it is important to limit the intake of foods that trigger hyperuricemia and hyperglycemia. Studying the effect of certain foods, such as seafood, on uric acid and glucose levels is critical.

Seafood serves as an excellent source of essential nutrients, including vitamins B12, D, and A, along with proteins, fatty acids, and minerals (Hu and Chan 2020). Shellfish offer notable health benefits, making it a highly attractive option when sourced from clean and uncontaminated water sources. It is low in calories, lean protein, and a major source of omega‐3 fatty acids.

Furthermore, clams, like other shellfish consumed in the Mediterranean diet, are a healthy and recommended food item (Xu et al. 2022). It stands out as a nutritious choice among shellfish, particularly in coastal regions. It is reported that the rich source of vitamins, microelements, and omega‐3 fatty acids in clams is extremely important for health (Plourde and Cunnane 2007) and can improve heart conditions due to its anti‐inflammatory properties. Its zinc levels can improve zinc deficiency, and this, together with omega‐3 fatty acids, promotes a healthy heart, brain, and immune system (Hosomi et al. 2012). Compared to oysters, clams have higher calories and protein content but less fat. Other micronutrients, such as selenium, represent 64% of the daily recommended value. Among its medical benefits, it has long been recognized for its anti‐tumor properties (Prescott et al. 1974).

Other studies have suggested that consuming clams (*Galatea paradoxa*), in moderation, will lower Low Density Lipoproteins (LDL), Triglycerides (TG), and cholesterol (Childs et al. 1990; Chijimatsu et al. 2009, 2011). Lin et al. (2022) also suggested its neuroprotective functions in fighting Parkinson's disease, which may be attributed to the presence of choline, an essential compound for neurotransmission (Haam and Yakel 2017). These benefits make clams an appealing food choice.

However, edible aquatic organisms may contain heavy metals such as cadmium, lead, mercury, and arsenic. In Ghana, shellfish such as clams, a freshwater bivalve, are harvested from the Volta River basin at locations such as Ada and Avaglo, near the Volta estuary, from March to September. A study by Obirikorang et al. (2010) on heavy metal concentrations in the Volta Basin identified mercury, manganese, and iron as present. The findings indicated that results were comparable to samples collected from low‐pollution areas. Thus, at the estuary, the metal levels do not exceed the clam's metabolic regulatory capacity. Clams used for this study therefore were safe for consumption.

Symptoms of allergy may also develop with the consumption of shellfish (Sicherer et al. 2004). These allergies may occur when the immune system reacts adversely to proteins found in the shellfish (Wai et al. 2020). Additionally, pathogens may thrive in shellfish if not well refrigerated (James et al. 2010).

Not many studies have been conducted to explore other adverse outcomes such as hyperuricemia with high clam consumption, even at the recommended daily allowance. The study aimed to determine how different levels of consumption of clams over a prolonged period would affect biochemical and hematological parameters.

## 2 Methodology

### 2.1 Sample Collection and Preparation

Fresh clams were obtained from market women who had just harvested the shellfish from a clean water body connected to the Volta River basin in the Ada area. These were stored in an ice chest and transported to the experimental site. The fresh clams were then placed in boiling water and shucked open to expose the meat. The clams were collected and progressively dried as follows: 80°C for 30 min, 100°C for 30 min; 100°C for 1 h, and 120°C for 1 h. Upon cooling, the meat was ground under sterile conditions using a mortar and pestle followed by further grinding into fine powder with a high‐speed electric grinder. The powder was sent for microbiological evaluation to assess microbial contamination. The product was certified as free of microorganisms.

### 2.2 Dose Selection and Justification

The doses used in this study (50, 150, and 250 mg/kg body weight) were derived from human dietary recommendations based on the US Food and Drug Administration (FDA) and Environmental Protection Agency (EPA) fish consumption advice of 8–11 oz per week (Voelker 2017). Using a representative portion of 3 oz (≈85.05 g) for a 70 kg adult yields a human equivalent of approximately 1214 mg/kg. Applying FDA‐recommended body surface area (BSA) conversion factors (*K*_m_ human = 37; *K*_m_ rat = 6) translates this to a theoretical rat equivalent dose of about 7500 mg/kg. However, because the present study utilized a concentrated extract rather than the whole food matrix, and to maintain a conservative safety margin consistent with exploratory preclinical studies, only a fraction (0.7%–3.3%) of the BSA‐scaled dose was administered. This range also reflects considerations of extracted yield, solubility, and tolerability observed in preliminary trials. Thus, the selected doses provide physiologically relevant exposure while ensuring animal welfare and compliance with ethical dosing limits. This approach aligns with FDA guidance for interspecies scaling (FDA 2005) and with standard preclinical practice where scaled‐down doses are used to approximate realistic human dietary intake while avoiding excessive pharmacological loading. It is also important to acknowledge that rat hepatic physiology differs from that of humans in both structural organization and enzymatic activity; nevertheless, these rats are used as models.

### 2.3 Animal Experimentation

Twenty‐eight (28) male Sprague Dawley rats, with weights between 200 and 250 g, were obtained from the Noguchi Memorial Institute for Medical Research (NMIMR). The rats were randomized and divided into four groups: three dose groups and a control group. The rats were housed under standard conditions in plastic cages with stainless steel covers and accommodated at an ambient temperature of 22°C ± 3°C and humidity of 40%–55%. The rats were kept under a 12:12 light–dark cycle throughout the experiment. Additionally, they were fed a standard chow diet and had access to drinking water ad libitum.

To minimize microbiome‐related variability, all animals were maintained on the same standard commercial chow diet and had no prior exposure to differing food sources before the commencement of the experiment. The animals were orally gavaged daily for 60 days. Furthermore, weekly weight measurements were obtained. All experimental rats were 6–8 weeks old at the start of the study, corresponding to young adult age.

Ethics clearance for the study was obtained from the University of Ghana Institutional Animal Care and Use Committee with ethics number UG‐IACUC 039/23‐24.

### 2.4 Sample Collection, Blood and Histopathological Analysis

On day 61, the experiment was terminated and rats anesthetized with 0.1 mL/100 g of bwt of Anaket (Neon Laboratories, India) and Chanazin (Chanelle Pharma, Ireland) (4:1) for blood and organ collection. Blood samples were drawn via cardiac puncture and transferred into heparinized and EDTA tubes for biochemical and hematological analyses. The liver function test comprised albumin, total protein, globulin, AG ratio, total bilirubin, direct and indirect bilirubin, AST, ALT, ALP, TBA. Renal function tests included blood urea nitrogen (BUN), creatinine, and uric acid. Other miscellaneous examinations included amylase, creatine kinase, and glucose. Lipid profile was also determined. Hematological analyses consisted of a full blood count. The rats were then euthanized using isoflurane (Pharmanova, India), and organs such as the liver, kidney, heart, and spleen were harvested. The organs were rinsed in normal saline and weighed. Samples for histology were stored in 10% buffered formalin for histological analysis.

Hematology samples for full blood count were immediately analyzed using a hematology Sysmex‐KX‐2IN hematology auto‐analyzer. Heparinized samples for biochemistry were analyzed using the Seamaty VG2 dry chemistry analyzer (Sichuan Province, China). Histological samples were later processed using the Leica TP 1020 tissue processor. The embedded tissues were sectioned at 4 μm thickness using a rotary microtome. These were later processed in an alcohol‐xylene series and subsequently stained with haematoxilin and eosin. The slides were later examined using an Olympus CX23 light microscope (Tokyo, Japan).

### 2.5 Statistical Analysis

Data was processed in SPSS version 29.0.2. Continuous variables were determined by ANOVA and expressed as mean ± standard deviation. Tukey post hoc multiple comparison test was used to determine where significant differences were observed in the specific groups of ANOVA. A *p*‐value ≤ 0.05 was considered significant.

## 3 Results

### 3.1 Toxicological Profile

Throughout the experimental period, none of the animals in any treatment group exhibited overt clinical signs of toxicity, including dullness, aggressiveness, sluggishness, alterations in locomotor activity, piloerection, excessive salivation, or lacrimation. Rats gained weight consistently (from 220–230 to 271–287 g) with no significant inter‐group differences. Relative organ weights showed no significant differences except for the liver, where weight decreased with dosage (significant between control and high dose, *p* = 0.007) (Table 1).

**TABLE 1:** Effect of clam on the relative organ weights of rats following 60 days' oral administration.

Organ	Control	LD	MD	HD	*p*
R. Heart	3.36 ± 0.5	3.26 ± 0.19	3.11 ± 0.18	3.33 ± 0.32	NS
R. Lung	6.73 ± 0.7	6.04 ± 1.66	6.79 ± 1.11	7.13 ± 1.8	NS
R. Liver	3.62 ± 0.1^a^	3.39 ± 0.23	3.40 ± 0.27	3.20 ± 0.2^a^	0.007^a^
R. Kidney (L)	3.13 ± 0.3	3.31 ± 0.25	3.12 ± 0.51	3.10 ± 0.3	NS
R. Kidney (R)	3.17 ± 0.3	3.35 ± 0.24	3.21 ± 0.15	3.05 ± 0.2	NS
R. Spleen	1.79 ± 0.2	2.15 ± 0.5	1.90 ± 0.20	2.01 ± 0.5	NS
R. Testis (L)	3.85 ± 3.6	7.28 ± 2.0	7.50 ± 0.90	7.36 ± 3.0	NS
R. Testis (R)	6.03 ± 4.3	7.57 ± 2.24	8.54 ± 1.04	8.44 ± 1.1	NS
R. Sem. vesicle	4.82 ± 1.5	4.75 ± 1.82	5.00 ± 1.02	5.24 ± 1.8	NS
R. Prostate	2.41 ± 0.7	2.27 ± 0.57	2.14 ± 0.73	2.08 ± 0.8	NS

*Note:* Values are expressed as mean ± standard deviation. *n* = 7 per group. Low dose 50 mg/kg bwt (LD), medium dose 150 mg/kg bwt (MD), and high dose 250 mg/kg bwt (HD).

Abbreviations: NS, Statistically not significant; R. Heart, Relative weight of heart; R. Kidney (L), Relative weight of kidney (L) %; R. Kidney (R), Relative weight of kidney (R) %; R. Liver, Relative weight of liver %; R. Lung, Relative weight of lungs %; R. Prostate, Relative weight of Prostate %; R. seminal vesicle, Relative seminal vesicle %; R. Spleen, Relative weight of Spleen %; R. testes (L), Relative weight of testes (L) %; R. testes (R), Relative weight of testes (R) %.

^a^ Control vs. high dose.

### 3.2 Liver Function Test

Significant differences were seen in globulin and A/G ratios, though not dose dependent. Total bilirubin significantly reduced in low (*p* = 0.031) and medium (*p* = 0.047) dose groups. AST levels varied significantly but without a clear dose–response; a difference was noted between MD and HD (*p* = 0.048). TBA increased significantly in the MD group (*p* = 0.000) but not in a dose‐dependent manner (Table 2).

**TABLE 2:** Effect of clam on liver function in rats after 60 days of oral administration.

Parameter	Control	LD	MD	HD	*p*
ALB	36.65 ± 1.6	34.98 ± 1.72	37.00 ± 0.5	34.71 ± 3.2	NS
TP	71.16 ± 2.0	69.8 ± 2.4	67.83 ± 0.5	69.06 ± 3.3	NS
GLOB	34.5 ± 1.6	34.83 ± 1.5^b^	30.83 ± 0.1^b^	34.32 ± 3.9	0.049^b^
A/G	1.06 ± 0.1	1.01 ± 0.06^b^	1.2 ± 0.00^b^	1.03 ± 0.2	0.036^b^
TB	4.03 ± 2.2^a^, ^c^	1.4 ± 1.3^a^	1.27 ± 1.03^c^	2.14 ± 0.9	0.047^c^; 0.031^a^
DB	1.44 ± 0.5	0.82 ± 0.8	0.93 ± 0.80	1.28 ± 0.3	NS
IBIL	2.54 ± 2.09	0.58 ± 0.9	0.33 ± 0.2	0.88 ± 0.7	NS
AST	151.7 ± 27.5	154.3 ± 20.03	203 ± 72.01^d^	132.2 ± 25.3^d^	0.048^d^
ALT	108.9 ± 39.3	84.67 ± 14.8	112.67 ± 22.2	84.6 ± 33.1	NS
ALP	514.7 ± 103.5	507.67 ± 102.3	518.33 ± 144.5	332.2 ± 182.9	NS
TBA	16.36 ± 3.9^a^, ^c^	26.8 ± 6.5^a^, ^b^	50.02 ± 9.5^b^, ^c^, ^d^	25.80 ± 5.8^d^	0.000^b^; < 0.0001^d^; 0.034^a^; < 0.0001^c^.

*Note:* Values are expressed as mean ± standard deviation. *n* = 7 per group. Statistically significant at *p* < 0.05. Low dose 50 mg/kg bwt (LD), medium dose 150 mg/kg bwt (MD) and high dose 250 mg/kg bwt (HD).

Abbreviations: A/G, albumin/globulin ratio; ALB, albumin (g/L); ALP, alkaline phosphatase (U/L); ALT, alanine aminotransferase (U/L); AST, aspartate aminotransferase (U/L); DB, direct bilirubin (μmol/L); GLOB, globulin (g/L); IBIL, Indirect bilirubin (μmol/L); NS, Statistically not significant; TB, total bilirubin (μmol/L); TBA, total bile acid (μmol/L); TP, Total protein (g/L).

^a^ Control vs. low dose.

^b^ Low dose vs. medium dose.

^c^ Medium dose and control dose.

^d^ Medium dose vs. high dose.

### 3.3 Lipid Profile

In Table 3, no statistically significant changes, though total LDL, and HDL cholesterol, but not increases with dose, were observed.

**TABLE 3:** Effect of clam extract on the lipid profile of rats after 60 days of oral administration.

Parameter	Control	LD	MD	HD	*p*
TC	1.44 ± 0.2	1.45 ± 0.2	1.55 ± 0.1	1.68 ± 0.2	NS
TG	1.08 ± 0.3	1.33 ± 0.3	1.34 ± 0.4	1.174 ± 0.4	NS
HDL	0.80 ± 0.1	0.75 ± 0.1	0.79 ± 0.06	0.83 ± 0.2	NS
LDL	0.15 ± 0.2	0.11 ± 0.2	0.25 ± 0.1	0.322 ± 0.2	NS

*Note:* Values are expressed as mean ± standard deviation. *N* = 7 per group. Statistically significant at *p* < 0.05. Low dose 50 mg/kg bwt (LD), medium dose 150 mg/kg bwt (MD) and high dose 250 mg/kg bwt (HD).

Abbreviations: HDL, high‐density lipoprotein (mmol/L); LDL, low‐density lipoprotein (mmol/L); NS, statistically not significant; TC, Total cholesterol (mmol/L); TG, triglycerides (mmol/L).

### 3.4 Renal Function

BUN, creatinine, and BUN/creatinine ratio showed no differences. Uric acid increased dose‐dependently: highest in the high‐dose group (*p* = 0.018 vs. control; *p* = 0.037 vs. LD) Table 4.

In other Biochemical Markers, additional parameters, including glucose, amylase, creatinine kinase, are presented in Table 7. Among these, only glucose showed a marked dose‐dependent increase with values for the control at 7.63 ± 1.95 mmol/L, LD = 8.26 ± 2.7 mmol/L, MD = 10.55 ± 2.3 mmol/L, and HD = 17.6 ± 4.9 mmol/L. Statistical differences were observed between the control and HD (*p* = 0.000), LD vs. HD (*p* = 0.000), and the MD vs. HD (*p* = 0.015).

**TABLE 4:** Effects of clam extract on renal function in rats after 60 days of oral administration.

Parameter	Control	LD	MD	HD	*p*
BUN	5.81 ± 0.4	6.00 ± 0.6	5.61 ± 0.2	5.494 ± 0.4	NS
CREA	27.57 ± 8.8	27.0 ± 9.3	33.6 ± 5.3	23.8 ± 11.7	NS
BUN/CREA	228.3 ± 67.1	265.5 ± 165.5	171.21 ± 26.9	289.47 ± 165.9	NS
UA	128.2 ± 52.7^a^	145.07 ± 71.6^b^	161.62 ± 132.8	339.58 ± 169.7^a^, ^b^	0.037^b^; 0.018^a^

*Note:* Values are expressed as mean ± standard deviation. *n* = 7 per group. Low dose 50 mg/kg bwt (LD), medium dose 150 mg/kg bwt (MD) and high dose 250 mg/kg bwt (HD).

Abbreviations: CREA, Creatinine (μmol/L); BUN, blood urea nitrogen; NS, Statistically not significant; UA, uric acid (μmol/L).

^a^ Control dose vs. high dose.

^b^ High dose vs. low dose.

### 3.5 Hematological Parameters

As seen in Tables 5 and 6, MCHC decreased dose‐dependently (*p* = 0.005). Platelet count decreased significantly (*p* = 0.014), lowest in MD. MPV and P‐LCR increased dose‐dependently, with significant differences between control and HD (MPV *p* = 0.009; P‐LCR *p* = 0.010). Total WBC count decreased significantly; doses showed varying effects, but all were lower than control. Eosinophils showed differences in a non‐dose‐dependent manner, with the highest level in the medium‐dose group, which was statistically different from the control group (*p* = 0.000), LD vs. MD (p = 0.000), HD vs. MD (*p* = 0.000). Monocytes showed significant differences; control 0.706 ± 0.23 vs. MD 0.29 ± 0.10 (*p* = 0.020), while neutrophils showed a marked decline (Table 6).

**TABLE 5:** Effects of the clam extract on red blood cell indices in rats after 60 days of oral administration.

Parameter	Control	LD	MD	HD	*p*
RBC	8.02 ± 0.66	8.49 ± 0.44	7.56 ± 1.07	7.71 ± 1.13	NS
HGB	13.71 ± 1.11	14.43 ± 0.97	12.74 ± 2.11	10.77 ± 4.73	NS
HCT	40.2 ± 3.66	43.78 ± 1.59	40.08 ± 5.49	41.23 ± 6.99	NS
MCV	50.09 ± 0.86	51.63 ± 2.78	53.12 ± 1.61	53.30 ± 2.57	NS
MCH	17.071 ± 0.25	17.03 ± 1.13	16.84 ± 0.84	13.30 ± 4.67	NS
MCHC	34.13 ± 0.50^a^	32.98 ± 1.66	31.68 ± 1.68	24.73 ± 8.31^a^	0.005^a^
PLT	884.285 ± 127.49^b^	838.50 ± 158.90	374.00 ± 263.70^b^	695.00 ± 420.74	0.014^b^
MPV	7.443 ± 0.39^a^	8.30 ± 1.09	9.70 ± 1.70	10.43 ± 1.82^a^	0.009^a^
PDW	14.943 ± 0.13	15.15 ± 0.17	15.30 ± 0.27	15.13 ± 0.33	NS
PCT	0.658 ± 0.09	0.69 ± 0.13	0.38 ± 0.28	0.76 ± 0.50	NS
RDW‐CV	15.529 ± 1.09	15.00 ± 0.74	15.64 ± 0.58	15.90 ± 0.22	NS
RDW‐SD	29.257 ± 2.17	29.05 ± 1.78	31.26 ± 2.15	31.87 ± 1.66	NS
P‐LCC	94.286 ± 22.68	142.00 ± 53.37	126.40 ± 96.46	263.67 ± 214.33	NS
P‐LCR	10.757 ± 2.49^a^	17.45 ± 8.31	30.70 ± 14.61	34.57 ± 14.44^a^	0.010^a^

*Note:* Values are expressed as mean ± Standard deviation. *n* = 7. Statistically significant at *p* < 0.05. Low dose 50 mg/kg bwt (LD), medium dose 150 mg/kg bwt (MD) and high dose 250 mg/kg bwt (HD).

Abbreviations: HCT, hematocrits (%); HGB, hemoglobin concentration (g/dL); MCH, mean corpuscular hemoglobin (pg); MCHC, mean corpuscular hemoglobin concentration (g/dL); MCV, mean corpuscular volume (fL); NS, Statistically not significant; PCT, Plateletcrit (×10^3^); PDW, platelet distribution width (fL); Platelet MPV, mean platelet volume (fL); P‐LCC, Platelet Large Cell Coefficient; P‐LCR, Platelet Large Cell Ratio; PLT, platelet count (%); RBC, red blood cell count (×10^6^); RDW‐CV, red cell distribution width coefficient of variation (%); RDW‐SD (fL), red cell distribution width‐standard deviation.

^a^ Control vs. high dose.

^b^ Control vs. medium dose.

**TABLE 6:** Effects of clam extract on white blood cell indices in rats after 60 days of oral administration.

Parameter	Control	LD	MD	HD	*p*
WBC	8.907 ± 3.25^a^	3.97 ± 2.98^a^	5.58 ± 2.40	6.37 ± 1.44	0.048^a^
Neu%	22.886 ± 7.38	24.03 ± 6.22	22.84 ± 8.34	25.30 ± 4.28	NS
Lym%	63.514 ± 11.16	58.68 ± 14.14	60.26 ± 13.79	63.07 ± 5.64	NS
Mon%	8.3 ± 1.76	11.35 ± 7.42	6.06 ± 2.88	5.70 ± 2.57	NS
Eos%	5.1 ± 2.99^c^	4.75 ± 1.90^d^	9.88 ± 3.49^b^, ^c^, ^d^	5.57 ± 1.96^b^	< 0.0001^b^, ^c^, ^d^
Bas%	0.2 ± 0.08	1.20 ± 1.10	0.96 ± 1.01	0.37 ± 0.12	NS
Neu#	1.87 ± 0.58^a^	0.84 ± 0.52^a^	1.15 ± 0.36	1.59 ± 0.25	0.013^a^
Lym#	5.916 ± 2.52	2.57 ± 2.18	3.58 ± 2.00	4.16 ± 1.15	NS
Mon#	0.706 ± 0.23^c^	0.38 ± 0.25	0.29 ± 0.10^c^	0.39 ± 0.25	0.020^c^
Eos#	0.399 ± 0.16	0.16 ± 0.10^d^	0.52 ± 0.26^d^	0.33 ± 0.06	0.027^d^
Bas#	0.017 ± 0.00	0.03 ± 0.01	0.04 ± 0.02	0.02 ± 0.01	NS

*Note:* Values are expressed as mean ± standard deviation. Low dose 50 mg/kg bwt (LD), medium dose 150 mg/kg bwt (MD) and high dose 250 mg/kg bwt (HD).

Abbreviations: Bas, Basophils count (U/L); Eos, Eosinophils (U/L) count; Lym, lymphocytes; Mon, Monocyte (U/L); Neu (%), Neutrophil (%); Neu, Neutrophils count (U/L); NS, statistically not significant; WBC, white blood cell count (×10^3^).

^a^ Control vs Low dose.

^b^ High vs. medium dose.

^c^ Control vs. medium dose.

^d^ Low dose vs. medium dose.

## 4 Discussion

The study dose range (50, 150, and 250 mg/kg body weight) was informed by national dietary guidance and scaled conservatively for preclinical testing. For transparency: a representative human portion of 3 oz (≈85.05 g) for a 70‐kg adult corresponds to ~1214 mg/kg. Using FDA BSA conversion factors (*K*_m__human = 37; *K*_m__rat = 6), the theoretical rat equivalent of this portion is ≈7500 mg/kg. Because our experiments used a concentrated aqueous extract, and because pilot tolerability and formulation/solubility constraints limited achievable intragastric/intraperitoneal concentrations, we selected a conservative experimental range of 50–250 mg/kg (≈0.7%–3.3% of the literal BSA‐scaled whole‐food equivalent). This approach is consistent with first‐in vivo exploratory studies where safety margins and extract concentration (rather than whole‐food mass) guide dose selection (Food 2017). The BSA calculation and rationale are shown for transparency; we note that full pharmacokinetic bridging (plasma exposure) or testing at higher doses would be required to claim strict quantitative equivalence to the human portion. It is important to note that rat hepatic physiology differs from that of humans in both structure and enzymatic activity. These species‐specific differences may influence xenobiotic metabolism and should be considered when extrapolating the present findings to human physiology.

From Table 1, the relative organ weights assayed (Heart, Lungs, Liver, Kidney [L], Kidney [R], Spleen, Testis [L], Testis [R], Seminal Vesicle, and Prostate) showed no statistical difference, except for the liver. A significant decrease was observed in the high dose (*p* = 0.007) when compared to the control and high‐dose groups. The significant decrease may be related to the extract's potential hepatoprotective effects or necrosis (Hsu et al. 2010). Prostate weight also showed a dose‐dependent decrease across all groups, from the control to the high‐dose group. This decrease could be due to the extract's ability to reduce enlargement and its potential protective function (Torfadottir et al. 2013).

**FIGURE 1:**
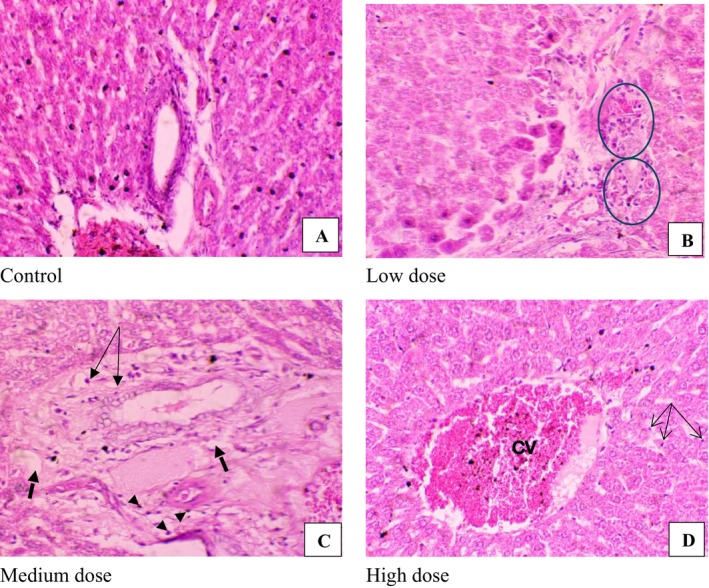
Micrographs of liver following 60 days of oral administration of clam extract. (A) Control group showing normal liver architecture. (B) The circled area shows infiltrates, possibly indicating inflammation. Note the proximity to the portal area suggesting hepatitis. (C) The arrows point to areas of proliferation of fibrous tissue, suggestive of liver damage in the form of fibrosis. Furthermore, proliferation of bile ducts is seen (arrow heads). (D) Inflammatory cells can be seen aggregating (arrows), while the central vein appears congested.

Liver function showed some changes in globulin, which consequently affected the albumin‐to‐globulin ratio, but not in a dose‐dependent manner. Similarly, changes in AST were not in a dose‐dependent manner. AST itself is not a specific liver biomarker compared to ALT (Table 2). Examining the profile may not clearly reveal any liver disease due to clam administration. However, the fairly dose‐dependent decrease in liver weight prompted the examination of liver tissue. Histologically, some deterioration was observed (Figure 1).

The irregularity of the non‐dose‐dependent analytes may suggest inconsistent absorption rates, saturation of the metabolic pathway, or altered excretion. Rats also display distinct hepatic enzyme ratios and metabolic sensitivities compared with humans, and their livers demonstrate high adaptability to xenobiotic exposure. These characteristics may influence the metabolism of dietary compounds and should be considered when interpreting the results.

As shown in Table 3, none of the lipid profile parameters differed significantly among treatment groups, indicating that clam extract consumption did not adversely affect lipid metabolism. In contrast, some hematological indices (Table 5) demonstrated statistically significant changes at the highest dose, specifically mean corpuscular hemoglobin concentration (MCHC; *p* = 0.005), mean platelet volume (MPV; *p* = 0.009), and platelet large cell ratio (P‐LCR; *p* = 0.010). These variations, although statistically significant, remained within normal physiological limits and are unlikely to represent treatment‐related toxicity. Similarly, white blood cell count (*p* = 0.048) and neutrophil percentage (*p* = 0.013) in Table 6 showed moderate elevations, which may reflect a mild physiological or immune response to repeated exposure rather than a pathological process.

The absence of consistent or dose‐dependent alterations across other parameters suggest that the extract did not induce systemic or organ‐specific toxicity at the administered doses. Reporting both significant and non‐significant findings provide a complete toxicological profile and ensures interpretive balance. In line with the OECD (Guideline 407) and ARRIVE 2.0 recommendations, comprehensive presentation of all measured outcomes enhances transparency, reproducibility, and the scientific integrity of the study. Standard laboratory chow does not contain fruits or green leafy vegetables, which in human diets provide alkalinizing effects that may help regulate uric acid and glucose metabolism. This dietary difference may partially account for variations between rodent and human metabolic responses.

Renal function was largely normal, except for uric acid, which increased in a dose‐dependent manner. Therefore, the rats handled the extract in a consistent mechanism and with pathways that led to predictable results (Table 4). Similarly, glucose concentration increased in a dose‐dependent manner (Table 7). These two analytes, uric acid and glucose, have a unique metabolic relationship. Increasing uric acid levels in the body results in the increase of pro‐inflammatory markers including IL 6 and CRP (Kang et al. 2005). Subsequently, there is a decrease in insulin sensitivity (Chaudhary et al. 2013) resulting in an increase in blood glucose. Rats possess limited renal excretion capacity for uric acid and rely partly on enzymatic degradation pathways, unlike humans who excrete uric acid directly. This species difference is an inherent limitation of rat‐based hyperuricemia models.

**TABLE 7:** Effects of the clam extract on glucose, amylase, and creatine kinase of rats after 60 days of oral administration.

Parameter	Control	LD	MD	HD	*p*
GLU	7.63 ± 1.95^a^	8.26 ± 2.7^c^	10.55 ± 2.3^b^	17.60 ± 4.9^a^, ^b^, ^c^	0.000^a^; 0.000^c^; 0.015^b^
AMY	2110.6 ± 103.8	2081.3 ± 280.5	1975 ± 51.4	1899.6 ± 190.3	NS
CK	383.4 ± 200.2	302.3 ± 90.99	586.33 ± 526.6	519.4 ± 755.8	NS

*Note:* Values are expressed as mean ± standard deviation. *n* = 7 per group. Statistically significant at *p* < 0.05. Low dose 50 mg/kg bwt (LD), medium dose 150 mg/kg bwt (MD) and high dose 250 mg/kg bwt (HD).

Abbreviations: AMY, amylase (U/L); CK, creatine kinase (U/L); GLU, glucose (mmol/L); NS, Statistically not significant.

^a^ Control vs. high dose.

^b^ Medium dose vs. high dose.

^c^ Low dose vs. high dose.

The emergence of hyperuricemia as a trigger for metabolic disease, along with its subsequent role as a risk factor for obesity, diabetes, hypertension, and cardiovascular disease, has been documented (Friedman et al. 2018). Hyperuricemia drives intestinal barrier dysfunction through gut microbiota dysregulation (Lv et al. 2020).

Although uric acid is an antioxidant, it also exhibits pro‐oxidant properties, leading to oxidative stress, which can affect insulin gene expression, resulting in decreased insulin secretion (Matsuoka et al. 1997). Cellular damage may also result from the activation of inflammatory factors. In addition, the insulin pathway is inhibited by high uric acid levels. Furthermore, there is the triggering of this pathway by ectonucleotide pyrophosphatase/phosphodiesterase (ENPP1) recruitment. This functions through its receptors (Tassone et al. 2018).

The relationship between uric acid and glucose remains a topic of debate. Large‐scale research by EPIC involving a cohort of 10,576 participants with a follow‐up period of 10 years supports this claim. Higher uric acid levels were associated with a higher risk of diabetes after adjusting for confounders. The hazard ratio was calculated as 1.20 (Sluijs et al. 2015). Other studies have also reported that elevated uric acid levels resulted in an increased risk of impaired fasting glucose (IFG) and the possibility of developing metabolic syndrome (MetS) (Anothaisintawee et al. 2017; Bombelli et al. 2018).

In this study, there was a significant decrease in liver size (Table 1). The histology does suggest some degree of fibrosis. The link between hyperuricemia and fibrosis has been reported elsewhere (Xiong et al. 2019). Some mechanisms have been proposed. Hyperuricemia leads to the production of pro‐inflammatory cytokines, such as IL 1β and IL 18, subsequently causing necrosis and apoptosis (Friedman et al. 2018; Castera et al. 2019; Swanson and Deng 2019). This scenario leads to ROS production, possibly due to hyperuricemia, resulting in enhanced xanthine oxidase activity and excess superoxide production, which subsequently triggers fibrogenesis mediators from inflammatory and Kupffer cells. This process invokes hepatic stellate cells (Sanchez‐Valle et al. 2012), leading to fibrosis.

Furthermore, these liver changes may have extended to the bile duct, leading to bile duct proliferation (Figure 1) and possible cholestasis. However, liver function tests did not suggest such underlying processes, except for the obvious changes in bile acid. Indeed, Table 2 demonstrated significant increases in bile acid compared to the control group. Other researchers also reported that bile acid levels were abnormal in hyperuricemia, based on an animal model. Notably, lowering uric acid with allopurinol improved lipid metabolism, which was accompanied by alterations in gut microbial composition in that study. It was observed that, due to intestinal barrier dysregulation and gut microbiota changes, the application of allopurinol lowered uric acid levels and altered microbiota composition (Lv et al. 2020).

The pursuit of a healthy lifestyle has driven many to seek Mediterranean‐like diets, especially among diabetes patients (Martín‐Peláez et al. 2020) and has also generated overwhelming interest among researchers (Yan et al. 2024). However, it must be noted that seafood, including shellfish, may trigger hyperuricemia due to its high purine content (Xu et al. 2022).

## 5 Conclusion

The findings in this study indicate a potential link between prolonged clam consumption and metabolic disturbances characterized by elevated uric acid and glucose levels. Given the increasing reliance on seafood as a dietary protein source, these results underscore the need for cautious dietary monitoring and further epidemiological studies in humans to clarify the long‐term health risks. Understanding this relationship has important public health implications for dietary guidance and the prevention of metabolic disorders associated with excessive purine and nutrient intake.

## Author Contributions

**George A. Asare:** conceptualization, resources, supervision, formal analysis, writing – original draft. **Samuel Adjei:** supervision, resources, formal analysis, writing – original draft, review and editing. **Matilda Asante:** writing – review and editing. **Charles Mills‐Robertson:** animal experiments, data curation. **Perpetua Dagadu:** supervision, animal experiments, data curation. **Stephen Yormasah:** statistical analysis. **Ruth Owu:** writing – review and editing. **Samkeliso Takaidza:** data analysis. **Vivash Naidoo:** histological analysis.

## Funding

The authors have nothing to report.

## Conflicts of Interest

The authors declare no conflicts of interest.

## Data Availability

The data that support the findings of this study are available from the corresponding author upon reasonable request.
